# Production and Optimization of Conjugated Linoleic and Eicosapentaenoic Acids by *Bifidobacterium lactis* in Cold-Pressed Soybean Cake

**DOI:** 10.3389/fnut.2022.916728

**Published:** 2022-07-27

**Authors:** Samin Rafi Azari, Mohammad Hojjatoleslamy, Zeinab E. Mousavi, Hossein Kiani, Sayed Mohammad Ali Jalali

**Affiliations:** ^1^Department of Food Science and Technology, Shahrekord Branch, Islamic Azad University, Shahrekord, Iran; ^2^Bioprocessing and Biodetection Laboratory, Department of Food Science and Engineering, Campus of Agriculture and Natural Resources, University of Tehran, Karaj, Iran; ^3^Department of Animal Sciences, Faculty of Agriculture and Veterinary Medicine, Shahrekord Branch, Islamic Azad University, Shahrekord, Iran; ^4^Research Center of Nutrition and Organic Products, Shahrekord Branch, Islamic Azad University, Shahrekord, Iran

**Keywords:** conjugated linoleic acid (CLA), EPA, *Bifidobacterium lactis*, soybean pressed cake, RSM

## Abstract

**Background and Purpose:**

In regard to the biosynthesis of conjugated linoleic acid (CLA) and eicosapentaenoic acid (EPA) by some bacteria, the objective of this study was to evaluate the efficiency of solid-state fermentation based on soybean pressed cake (SPC) to produce CLA and EPA by *Bifidobacterium lactis*. The objective of this study was to evaluate the efficiency of solid-state fermentation based on SPC to produce CLA and EPA by *B. lactis*.

**Methods:**

Process conditions including humidity, inoculation level, and temperature parameters were optimized by adopting the response surface methodology (RSM) method (response surface method) and the design expert software. Accordingly, a homogeneous SPC paste substrate at 60, 70, and 80% humidity was prepared with different inoculation levels at 30, 37, and 44°C to assess the strain behavior. The introduced SPC consisted of 60% humidity, 2% inoculation level at 37°C, and 60% humidity, and 4% inoculation level at 30 and 44°C; it also included 6% inoculation level at 37°C, 70% humidity at 2% inoculation level, at 30 and 44°C, and 4% inoculation level at 37°C. Also, SPC with 80% humidity at 2% and 4% inoculation levels, and at 30 and 44°C was obtained. To confirm the accuracy of the conditions, an experiment was conducted according to the defined requirements.

**Results:**

The results were compared with the predicted data, which showed a significant difference. Under optimized conditions, with an inoculation level of 4% on the SPC medium with 70% humidity and at 37°C, *B. lactis* strains could yield 9*cis-*, 11 *trans-*linoleic and eicosapentaenoic at 0.18 and 0.39% of the total fatty acids.

**Conclusion:**

So, the potential benefits of using SPC as an inexpensive substrate for the commercial production of CLA and EPA should be noted.

## Introduction

Conjugated linoleic acid (CLA) consists of a set of positional and geometric isomers of octadecadienoic acid (LA) with a conjugated double bond bonding system (–C=C–C=C–) initiated from 9, 10, or 11 carbons. All settings of *cis-*trans, *trans-*cis, *cis-cis* and *trans-trans* are possible in each of the three positional systems (1–3). There exist at least 28 known CLA isomers (4), among which *cis* 9 and *Trans* 11 (mostly in meat and dairy) have anti-cancer properties. *Trans* 10 isomer, *cis* 12 (mostly in vegetable oil) (5), is active in the body’s fat reduction and weight loss, as well as the increase of energy consumption (6, 7). It is the most abundant and important in terms of naturalness, with about 85 and 10%, respectively (1, 8).

On the other hand, CLA is a natural unsaturated *trans* fatty acid (1) available in the meat and milk fat of sheep, goats and deer (1, 9–10). Despite being a specific *trans* fatty acid, it is categorized as non-trans, according to FDA; it is also known as a safe GRAS (7, 11–13). In addition, eicosapentaenoic acid (EPA) is one of the most unsaturated fatty acids of omega-3 type with a potentially beneficial effect on the cardiovascular system (14) in different volumes, within the range of 0.1 to 2.6 g in some fish types, especially cold-water fish. It is found in a group of seaweed. A group of bacteria like *gamaproteobacteria* and *schwannellas* can biosynthesize this fatty acid (15). Although EPA is a nutrient and is produced as a supplement, it is also called a drug because of its specific function and result. Studies show that taking EPA supplements in people with high blood triglyceride levels can reduce the level of this fat by up to 33% (16).

In the recent years, more attention has been paid to the production of healthy and safe food products because consumers are looking for more natural foods (17) to improve their health through its active ingredients (13). Fats are one of the main components of foods; due to their association with cardiovascular diseases, diabetes and obesity, there are many concerns about the type and amount of their consumption, because the quality of fat in the diet (9) is of great importance.

Despite the anti-nutritional nature of some lipids (6), such as *trans* and saturated fatty acids, another group of lipids has shown beneficial physiological effects. In this regard, fatty acids have received much attention due to their positive effect on the prevention of a number of diseases (7, 9). For this reason, the necessity of using biotechnological ways to produce healthy fats has attracted much attention, with a good potential in terms of producing safe and healthy fat products, as shown in the studies focused on producing and incorporating them into foods (4, 18, 19).

In this context, one of these health-promoting or pragmatic lipids is conjugated linoleic acid (CLA), the isomer of CLA 9-cis, 11trans, which has a potentiating effect on the transmission of PPARγ (fatty acids) nucleus, the main regulator of fat cell differentiation. It acts as a stimulant of adiponectin secretion, and this mechanism can partially counteract the anti-hypertensive, anti-hyperlipidemic, anti-angiogenic effects (effective in preventing cancer metastasis), as well as offering atherosclerotic, anti-cancer and anti-diabetic properties helpful for improving the human’s health (1, 7, 10).

In contrast, the CLA isomer, trans10, could increase cis12 lipolysis, reducing the function of fatty acid synthesis (20), as well as being associated with proatrogenic effects, insulin resistance, and inflammation (5, 17). In addition, CLA has other beneficial physiological effects, such as increasing body muscle, improving the immune system, providing antioxidants (free radical scavengers), serving as an anti-allergy agent and reducing platelet coagulation (2, 7, 18, 21).

The development of economic technologies to increase the nutritional value and bioactive compounds of natural resources (such as cereals and grains) has attracted considerable attention in the recent years (22). Every year, large quantities of residues are produced in the agricultural and food industries; if the recycling of this waste is well managed, it could have many economic and environmental benefits (23). In addition, the use of metabolites can generate new sources (24, 25). The waste of food oil factories is a problem in developed countries; it is of particular importance to researchers due to environmental issues (26) as the seeds of oil products are a significant share the production. So, it is considered as a source. Moreover, for proper evaluation, waste recycling can be important in terms of economic, environmental, social and ecological aspects. Therefore, conversion of biomass into high value-added compounds can be very beneficial (23). As the use of agricultural waste in industry also reduces production costs, it usually accounts for 25 to 50 percent of total production costs (7). Its consumption improves various biological parameters related to the human’s health.

Soybean is the most commonly produced oil crop in the world. Soybean oil is primarily used in the production of shortening, margarines, cooking/frying oils, salad dressings, and mayonnaise (27). Soybeans are currently one of the most important foods (28); they are the second largest source of vegetable oil worldwide (after palm oil), with a high economic value (29). They also have a strategic potential in food safety and bioactive compounds for human needs (30). In addition, they could be regarded as one of the most popular plant foods used in food and medicine. Soybeans are mechanically pressed to extract oil by cold pressing or chemically processed with organic solvents such as hexane. The cold press method leads to products free of organic solvents (31). After the production of oil from oil seeds, valuable by-products (cakes/meal) rich in proteins, few lipids, carbohydrates, and bioactive compounds may be obtained (30, 32, 33). In addition, they could be considered as a rich source of protein, as they contain amino acids, oligosaccharides, vitamins B and E, and minerals (34). Also, they contain isoflavones that can promote the growth of microorganisms. Moreover, soybean mills can use them as a substrate in biological fermentation processes to produce fatty acids (35). Soybean cake is an important source for bioactive compounds such as phenolic compounds and lecithin which are proved that have health benefits (27). In addition using this waste can help the environmental condition and providing huge economic benefits (36).

One of the oldest processes applied by humans is the solid state fermentation used for food (11). Solid fermentation is a biological process with a high potential for bio-enhancing the conversion of plant wastes into many valuable compounds (22). Solid state fermentation has many advantages, including cost-effectiveness, low water consumption, less volume of equipment, high efficiency production per unit volume and easier aerobic process. In addition, there is an increase of the oxygen diffusion rate in wet solids (37, 38). The selection of bacteria capable of synthesizing CLA or CLNA under *in vitro* condition is the first step required to evaluate the possible effect of their production during food fermentation or their impact on the surface of the intestine. Conversion and isomer patterns depend on various factors (37). In this regard, bifidobacteria have attracted much attention; they are considered as the most interesting genus for the in-situ production of CFA (6). There is also some evidence showing that the ability to convert linoleic acid (LA) to CLA is strain specific, (5) and that the conversion rates vary, depending on growth conditions and matrix (39). Few studies have been, however, performed on the production of fatty acids with the help of a group of bacteria such as *Bifidobacterium* and *Lactobacillus* species, which are mainly used as probiotics (18). Most strains of *Bifidobacterium* are more efficient at synthesizing CLA and conjugated linolenic acid (CLNA), despite producing lower levels of conjugated fatty acids (37, 40).

Not only do they have the potential to accelerate the production or recovery of conjugated linoleic acid and CLNA from LA and α-linolenic acid (ALA) (6, 20, 21, 34, 37, 39, 40), but also have the ability to grow in soybean litter and use its carbohydrates (sucrose, raffinose and stachyose) by modifying the substrate to increase its nutritional and functional properties (13, 34, 41). By producing the enzyme α-galactosidase or β-glucosidase and also, hydrolyzing the proteins in it, lactic acid can be produced with a decrease in pH. The capability of some species of LAB, including propionibacteria and bifidobacteria, to in-vitro conjugate LA or LNA has been considered for many years. Producing functional foods enriched in conjugated fatty acids by using them as starter or adjunct culture can be considered a promising topic for further development and study (39). Innovation has always been the key to success. We should make the optimal use to ensure future progress and success. Given to the rate of obesity and mortality due to cardiovascular diseases, diabetes and cancer (silent death), especially in the young generation (2), soybean meal can be considered as an excellent, natural, low-cost and cost-effective substitute serving as a substrate for use in solid state fermentation (SSF) for the production of fatty acids (7, 13, 20). The aim of this study was, therefore, to produce beneficial fatty acids (CLA) and (EPA) by *Bifidobacterium lactis* on a soybean meal-based substrate as a natural, rich, suitable, inexpensive and available environment to return part of the waste of food oil factories in the production cycle.

## Materials and Methods

### Materials

*Bifidobacterium lactis* (BBo4, Persian TypeCulture Collection) was provided from the Microbial Collection of the Microbiology Laboratory, Department of Food Science and Engineering, University of Tehran, Iran. The SPC and chemical solutions were provided from Ghiam Kesht and Sanat Company (Iran) and Merck (Germany), respectively.

#### Preparation of the Bacteria

**FIGURE 1:**
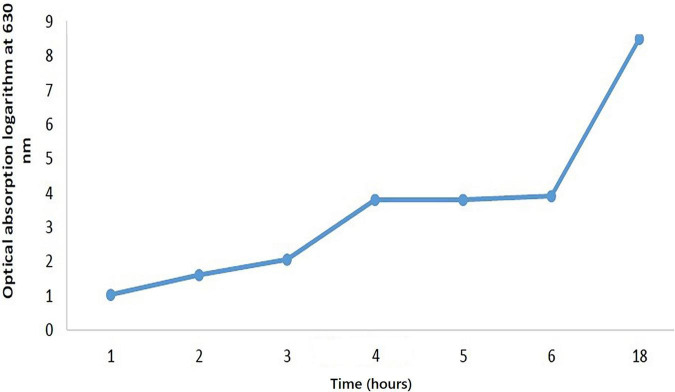
*Bifidobacterium lactis* growth curve.

In this process, the bacterium was linearly cultured twice, each for 24 h, in an MRS agar medium containing 0.5 gram per liter L-cysteine and placed in an anaerobic incubator (Model D-91126, Memmert Co., Germany) at 37°C. To enrich bacteria, they were first put in an MRS broth medium and incubated three times, each for 24 h, at 37°C. The cells were collected at the end of the growth phase in the MRS broth through the centrifuge and rinsed twice to obtain 1.5* 10^7^ in 0.5 Mcfarland standard; then the tirbidity of the batcerial suspension was adjusted to the 0.5 Mcfarland standard (1.5* 10^8^). The inoculation size was optimized to support solid bed fermentation; thus, dilutions of 2, 4, 6% were made. To draw the growth kinetics, curve was made by the first degree equation (rx = dCx/dt = μCx-KdCx) (41, 42). After bacteria transfer to the broth media, the agar rate absorption at 0, 1, 2, 3, 4, 6, and 18 h was measured to draw the curve (Figure 1).

#### Substrate Preparation

The SPC was taken out of the refrigerator and rinsed twice with water; then it was soaked in distilled water for 12 h. In the last stage, the surface water was wholly discarded, and SPC was rinsed again and poured in a mixer to be homogenized. About 150 g of this homogenized soybean paste was weighed by a digital scale of (0.0001 g) high accuracy that was put into a flask and sterilized by autoclave (Iran Tolid Medical Industries Co.) at 121°C for 15 min. After cooling, humidity was measured in the oven at 105°C; the humidity content of the meal was calculated as the base humidity.

#### Inoculation of Bacteria Into the Culture Medium

When the sterilized homogenized SPC reached the room temperature, the conjugated mixture without inoculation was considered as the control. Without adding any additives like vitamin k, hemein and sucrose, the microbial suspension was inoculated at 2, 4, and 6%. After that, it was homogenized (13) at three humidity levels (60, 70, and 80%) and poured on the plates; then they were incubated at 30, 37, and 44°C for 48 h as the response surface methodology (RSM) response levels (Table 1). The cells volume after inoculation was about 3, 6, and 9*10^7^ CFU/g, with 2, 4, and 6% inoculation, respectively. After 48 h, to ensure the purity of the inoculated bacteria, the slide was prepared and colored through the gram method; this was observed through the Olympus optical microscope, thus confirming the purity of bifidobacteria.

**TABLE 1:** Independent variables and response data in the response surface method (RSM) method.

Run	Real values (coded)			Response 1 = pH at 48 h	Response 2 = Absorbance
	X1	X2	X3		
1	37 (0)	60 (−1)	2 (−1)	5.5	0.2
2	44 (1)	70 (0)	2 (−1)	5.3	0.5
3	44 (1)	60 (−1)	4 (0)	5.4	0.45
4	37 (0)	80 (1)	2 (−1)	5.45	0.43
5	37 (0)	60 (−1)	6 (1)	5.35	0.55
6	30 (−1)	60 (−1)	4 (0)	5.41	0.51
7	44 (1)	80 (1)	4 (0)	5.48	0.5
8	37 (0)	80 (1)	6 (1)	5.1	0.7
9	30 (−1)	70 (0)	2 (−1)	5.2	0.72
10	44 (1)	70 (0)	6 (1)	5.3	0.67
11	30 (−1)	80 (1)	4 (0)	4.68	0.9
12	30 (−1)	70 (0)	6 (1)	4.64	1.2
13	37 (0)	70 (0)	4 (0)	4.3	2.2
14	37 (0)	70 (0)	4 (0)	4.2	2.15
15	37 (0)	70 (0)	4 (0)	4.15	2.21
16	37 (0)	70 (0)	4 (0)	4.1	2.25
17	37 (0)	70 (0)	4 (0)	4	2.24

*X1: Temperature, X2: Humidity, X3: Inoculation.*

### Microbial Count

Cell viability was determined through the dilution surface method in the MRS agar. After the inoculation of the solution, ten tubes were prepared. Each tube was placed on the MRS agar plate in the incubator for 24 h at 37°C; the results were expressed as CFU/g (13).

### Measuring pH Changes

The pH changes of the samples after inoculation time (0, 24, and 48 h) were recorded by a pH meter (Model 826 Metrohem Co., Swiss) and reported as Response 1 (7, 13), as can be seen in Table 1.

### Measurement of Conjugated Linoleic Acid by Spectrophotometer

After 48 h incubation, rapid spectrophotometry based on UV absorption was used for bifidobacteria screening and determination of their growth ability, producing CLA according to (5). The culture sample was centrifuged through the refrigerated centrifuge (Model 3-30K, Sigma Co., Germany) at 13,000 rpm; then 1 ml of the supernatant was mixed with 2 ml of isopropanol and vortexed after adding 1.5 ml hexane. Subsequently, the sample was held for 5 min at room temperature and the hexane layer was collected. Then the absorption rate was determined by a spectrophotometer (Biochrome WPA, United Kingdom) at 233 nm. The data were recorded as response 2 in the RSM model (Table 1).

### Fatty Acids Analysis

The modified Folch method was used for the lipid extraction of the SPC (20). Accordingly, 5 g of the sample was mixed and vortexed with a 100 ml 2:1 chloroform/methanol mixture; then it was centrifuged at 5,000 rpm and passed through Whatman paper No. 0.22. After that, 5 ml of distilled water was added to the filtered liquid, which was vortexed and centrifuged again for 10 min under the same conditions. At this stage, the upper phase was discarded and the lower phase was evaporated at 40°C and 80rpm by the rotary evaporator (IKA RV 10 model). The fatty acids composition of the sample was determined by the gas chromatography (GC) method (10); the National Standard of Iran No. 13126-2 was applied for the separation and methylation of fatty acids to produce fatty acid methyl ester (FAME). FAMEs were then separated and quantified in a gas chromatograph (Nexis 2030 Shimadzu model, Japan) equipped with a flame ionization detector (FID) and capillary column (Dikmacap-2330; 60 m × 0.25 mm × 0.20 μm), with the injector and detector temperature of 250°C and 260°C, respectively. The injection volume was 1 μl and hydrogen was used as the carrier gas with a flow rate of 2 ml/min. The column temperature, which began at 60°C, was held for 2 min. The temperature was raised to 200°C at a rate of 10°C min^–1^; then it was raised to a final of 240°C. This temperature was held for 7 min. The method of identifying the fatty acids was determined by comparison with the known mix fatty acids methyl standards. The data were presented as the percent of total fatty acids (TFA).

### Experimental Design and Statistical Analysis

The RSM method and the Box Behnken Design (BBD) were applied to predict and optimize the efficiency of a particular process and to maximize production through the response level method. Numerical optimization was also applied to obtain the appropriate answers. This design was actuated with three factors, each at three levels and with five repetitions in the center point, including 17 experimental tests. The homogeneous soybean meal pastes with 60, 70, and 80% humidity (X2) as the substrate and different inoculation values of 2, 4, and 6% (X3), at 30, 37, and 44°C (X1), were selected as the variables to assess the maximization of the bacterial strain response. In the RSM method, for each dependent variable, a model is defined, where the main and interacting factors on each variable are expressed (43, 44). The multivariate model was expressed through the Eq. 1.


(1) $$\text{Yn}=\mathrm{b0}+\sum_{\text{i}=1}^{3}\text{bixi}+\sum_{\text{i}=1}^{3}\text{biixij}+\sum_{\text{i}\neq \text{j}=1}^{3}\text{bijxixj}$$

where, Yn is the predicted answer, b0 is the constant coefficient, bi refers to the linear effects, bii is the quadratic effect, bij is the interaction, and xi and xj are the independent variables. Then the maximum and minimum limits of each variable were coded. The codes assigned to the independent variables are presented in Table 1. The optimal conditions were determined according to the response level method. The first and second factors (Temperature and Humidity: X1 and X2), without applying the third factor (Inoculation:X3), were involved in comparing the results. At this stage, the experiment was designed to allow the optimal conditions. The data were analyzed using the ANOVA Design Expert (Ver. 11) and SPSS (Ver. 16) software.

## Results and Discussion

### The Behavior of *Bifidobacterium* on the Substrate

The growth of *B. lactis* on the SPC is represented in Figure 1. To assess the growth pattern of microbes on this medium, the microbial population was compared with a specific culture of the MRS agar and the best growth pattern of microorganisms, in regard to the four variables during fermentation. So, pH, temperature and microbial growth stages are the most critical factors in the biological production and viscosity of inoculated linoleic acid (45). The growth of bifidobacteria on the SPC was evaluated in different conditions including pH, temperature, humidity and inoculum percentage.

The results showed that microorganisms could well grow in the substrate at different humidity, temperature and inoculum levels. At the beginning of the fermentation process, the bacterial population did not increase considerably. Due to the adaptation of the microorganisms to the new growth environment, there was no significant change in cell population and pH. However, after 18 h of fermentation, there was a sharp decrease in pH from 6.6 to 5.8. In addition, the cell population augmented the initial value. After 24 h, the bacterium entered the logarithmic phase, reaching its maximum population; the tarnished state due to this increase was evident on the medium. In the last h of fermentation (48 h), the decrease in the bacteria count was apparent by a lower drop of pH, at all humidity levels and 30 and 37°C, except 44°C. In contrast, the pH of the control sample remained unchanged. In general, the fermentation process of bifidobacteria lactis was within 8–48 h (7, 11, 40); however, in this experiment, the highest bacterial growth occurred within 24–48 h after inoculation. After the complete fermentation of the substrate, the bacteria growth rate in the SPC culture medium followed a descending trend. After approximately 48 to 72 h, it stopped, leading to the death of some bacteria. After 24 h of fermentation, no more change in pH was observed and a decrease in the decline of the cell population occurred. It was also found that with the increase of the fermentation time, the oligosaccharides and crude protein content of the soybean meal was consumed by bacteria, and solid-state fermentation could considerably increase the solubility of the protein in substrate amino acids (34). The hydrolysis of proteins in the fermented SPC depends on the type of bacterial strain and the substrate humidity content (42, 46). It was found that an increase in the produced lactic acid volume in the culture medium decreased the pH rate. Another influential factor is the inoculated bacteria level in their growth kinetics. Optimal bacterial growth depends on the internal and external humidity of the culture medium surface. Primary substrate humidity is a vital factor in bacterial growth, and a humidity level above 50% in SSF promotes the development of microorganisms (40, 45). After fermentation, the loss of high humidity content could lead to the increased material concentration at the culture medium, thus indicating the end of the growth phase (11). Bifidobacteria lactis in the SPC exhibited high compatibility at 70% humidity and 4% inoculation level, with the lowest pH of (4.0) at 37°C, (Table 1). After 48 h fermentation, no more change in pH was observed and a decline in cell population occurred. The bacteria count reduction rate after 48 h was due to the decrease in the pH and volume of the nutrients. No provision of the nutritional demand for microorganisms and low humidity could prevent their growth (11, 13, 40).

### Optimal Conditions Based on the Response Level

The results of the variance analysis of pH and absorption indices are tabulated in Table 2. The parameters R ^2^-sq, R ^2^ sq - (adj), and *p*-value indicated a high correlation between the observed and predicted values (47). Data analysis based on the linear model was, however, insignificant for all factors, and the most significant and high correlation model was the quadratic one. The high volumes of R ^2^-sq and R ^2^- adj for the models (98.72 and 97.07%) with pH and (98.82 and 99.6%) absorption response levels indicated that the predicted models for CLA and EPA production were suitable and of high regression coefficients. Lack of fit did not, however, show a significant effect. The first (*P* < 0.05) and second (*P* < 0.001) degree effects of the three parameters, namely, X1 (temperature), X2 (humidity) and X3 (inoculation), had a significant interaction with X1, X2 and X1 and X3 in terms of pH response absorption. Their interactions were significant at (*P* < 0.05) (Table 2).

**TABLE 2:** Analysis of variance related to pH and absorbance data obtained from the Box Behnken Design.

		Measurement	
Source	Degree of freedom	pH	Absorbance
Model	9	0.5633***	1.081633***
X_1_	1	0.3003***	0.183013***
X_2_	1	0.1128*	0.08405***
X_3_	1	0.1405**	0.201613***
X_1_X_2_	1	0.164**	0.0289*
X_1_X_3_	1	0.0784*	0.024025*
X_2_X_3_	1	0.01	0.0016
X_1_^2^	1	0.765***	1.827164***
X_2_^2^	1	1.87***	3.890533***
X_3_^2^	1	1.2***	2.55348***
Residual	7	0.0094	0.00247
Lack of Fit	3	0.0053	0.00369
Pure Error	4	0.0125	0.00155
Cor Total	16		
R^2^		98.72%	98.82%
R^2^ adj		97.07%	99.60%

***, **, *: Significant at p ≤ 0.001., p ≤ 0.01, p ≤ 0.05.

*X_1_ : Temperature, X_2_ : Humidity, X_3_ : Inoculation; MS: Mean Square.*

### pH Changes

**FIGURE 2:**
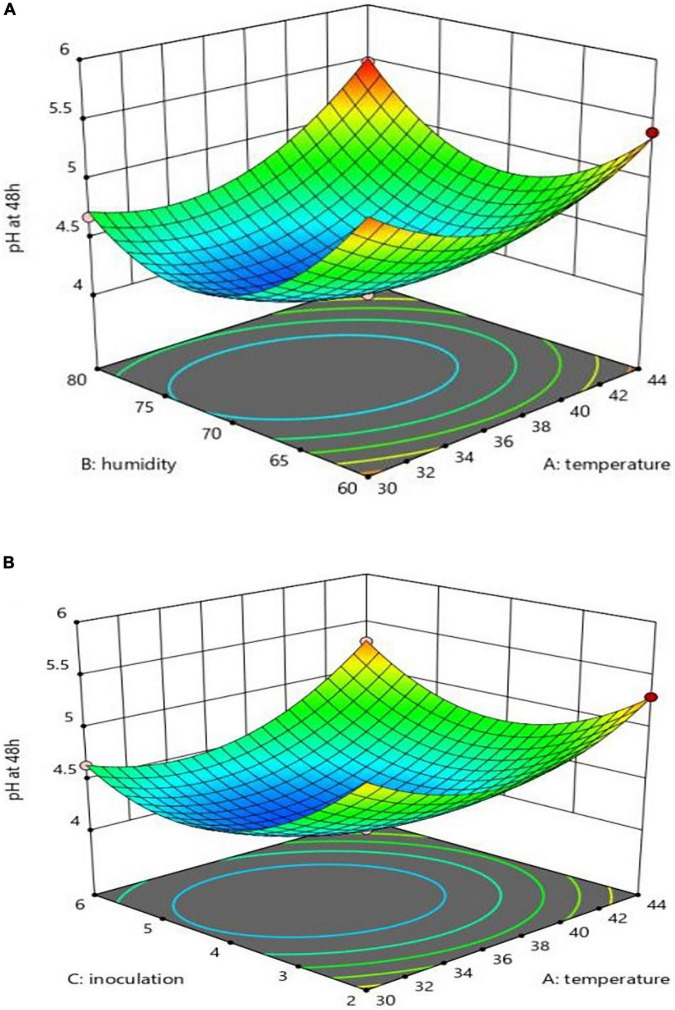
Interaction effect of temperature and humidity **(A)**, and temperature and inoculation **(B)** on pH.

Assessments run on the pH changes in each of the variables (48 h at the substrate) revealed that 44 degrees had a preventive effect on CLA production. The substrate pH was not reduced, as shown in Figure 2; meanwhile, the inoculation level had a positive impact, as compared to humidity, on increasing production (reduced pH), as can be seen in Figure 2. The interactions of X1 and X2 and X1 and X3 on production were, however, negative. Meanwhile, inoculation had no significant impact on humidity, as can be seen in Figures 2A,B. A separate analysis of the practical factors on the production yield showed an increase of temperature from 30 to 37°C, humidity from 60 to 70%, and inoculation from 2 to 4%. With the rise of temperature from 37 to 44°C (only after 24 h), humidity from 70 to 80%, and inoculation from 4 to 6%, the pH level followed a descending trend; after 48 h injection, the substrate pH remained unchanged at 44°C. The equation of pH changes based on these three parameters and their interactions, according to the significance of the coefficients of the equation, is expressed as follows:


(2) $$\begin{array}{c} \text{pH}=4.15+0.194{\text{X}}_{1}-0.119{\text{X}}_{2}-0.132{\text{X}}_{3}+0.426{\text{X}}_{1}^{2}\\ \;+0.666{\text{X}}_{2}^{2}+0.534{\text{X}}_{3}^{2}+0.203{\text{X}}_{1}{\text{X}}_{2}+0.14{\text{X}}_{1}{\text{X}}_{3} \end{array}$$

Note that because the interaction effect of X2 and X3 was insignificant, as shown in Table 2, they are not expressed in this equation.

The interaction diagram of X1_ X3 and X1 _X2 at 70% and the inoculation of 4% indicated that the highest pH was at 44°C and the inoculation of 6 and 2%, as shown Figure 2A; also, the temperature of 44°C and humidity of 80% should be mentioned, as can be seen in Figure 2B. For pH less than 4.2, the approximate inoculation range was estimated to be 3.5–5.0, and temperature was 31.9–38.9°C.

### Absorption

**FIGURE 3:**
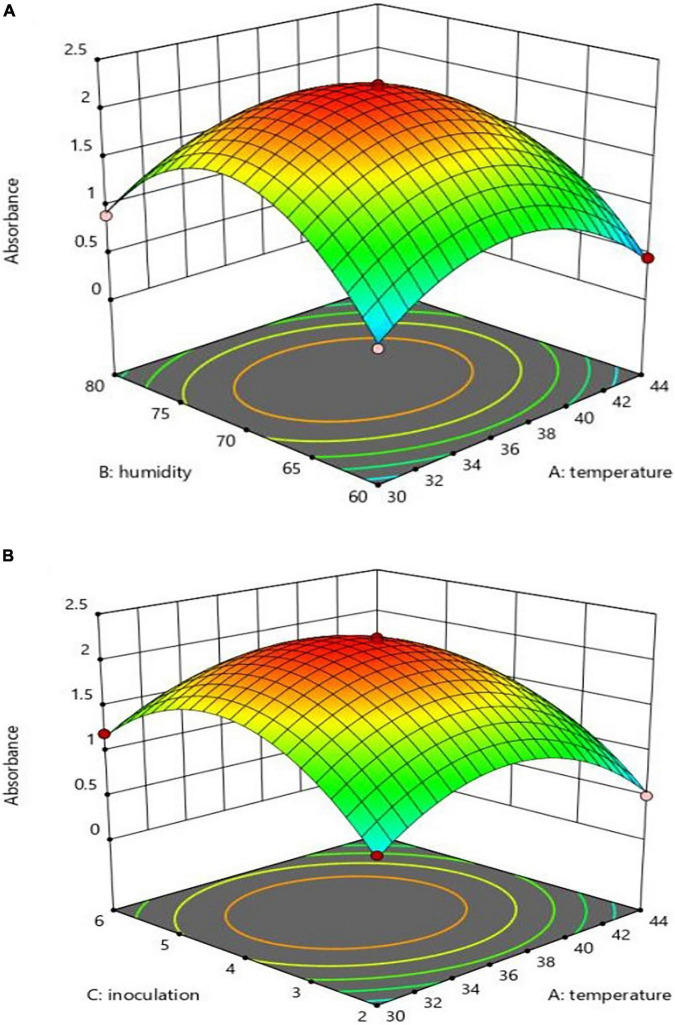
Interaction effect of temperature and inoculation **(A)**, and temperature and humidity **(B)** on absorption.

Assessing the variables’ absorption rate revealed that X2 had a more significant effect on the absorption rate than X1 and X3, while significant interactions could harm production. The 3D figures are based on the X1 and X3 interaction absorption rate, as shown in Figures 3A,B. The interaction effect of X1*X2 and X1*X3 on pH 48 h after inoculation revealed the highest absorption level results when pH was at its lowest, that is, X1 at 37°C, X3 at 4% and X2 at 70%, as can be seen in Figures 3A,B. Simultaneous optimization through the utility volume of 0.991% in the soybean meal was significant for all three responses. The correlations between the independent variables of the experiment and the absorption level concerning the insignificant nature of X2 and X3 interactions, according to Table 2, are expressed in Eq. 3:


(3) $$\begin{array}{c} \mathrm{Abs}.=2.21-0.151{\text{X}}_{1}+0.103{\text{X}}_{2}+0.159{\text{X}}_{3}-0.659{\text{X}}_{1}^{2}\\ -0.961{\text{X}}_{2}^{2}+0.779{\text{X}}_{3}^{2}-0.085{\text{X}}_{1}{\text{X}}_{2}-0.078{\text{X}}_{1}{\text{X}}_{3} \end{array}$$

### Bacterial Screening at the Production Rate

*Bifidobacterium lactis* was screened to produce EPA and CLA by measuring the absorbance at 233 nm. *B. lactis* in the SPC at different X1, X2, and X3 levels (except 44°C) 48 h after inoculation, at a decreased pH index, revealed the production ability of other volumes (42). The highest absorption rate (more than 2) in the culture medium was observed at 70% humidity, 37°C and 4% inoculation level, with a 4.15 pH level. At the temperature of 44°C, humidity of 80% and inoculation level of 6%, there was a negative effect on production, as also shown by (25). According to the optimal surface model, the highest production occurred in the central region, with the temperature of 36–37°C, inoculation level of 3.5–5%, and humidity of 68–72%. The highest optimum point of the predicted volume from the operations consisted of 36.040°C, 70.532% humidity, 4.222% inoculation and absorption point >2.231, as can be seen in Table 3. The experimental results, thus, confirmed the optimal rates. The substrate concentration strongly affected LA/LNA to CLA containing the produced oil conversion rate through the *B. lactis* strain (48). The CLA production was variable due to the response of bifidobacteria lactis to the substrate fatty acids level and composition (37). pH and temperature could be, therefore, regarded as important environmental parameters that change the structure and affect the diversity of distribution (49, 50).

**TABLE 3:** The optimal value of different independent variables (temperature, humidity, and inoculation) and responses based on the highest absorption rate at the lowest pH.

		Optimum value
Variable	Temperature	36.040
	Humidity	70.532
	Inoculation	4.222
Response	pH at 48 h	4.114
	Absorbance	2.231
	Appropriateness	0.991

### Fatty Acids Composition

The fatty acids (FA) profile of the SPC inoculated with (48 h) and without *B. lactis* and their changes are presented in Table 4. The most positive changes of FAs were seen in the two main strains of saturated FA (palmitic (C16:0) and stearic (C18:0) acids, with the inoculation of Bifidobacteria on the SPC. On the other hand, the mono and polyunsaturated FA (oleic (C18: 1n-9) and linoleic (C18: 2n-6) acids, which had the highest proportion of FAs in the SPC, showed the most negative change by bacterial inoculation (Table 4). *B. lactis* is a bacterial strain that can easily adapt itself to using substrate nutrients (37), such as FAs, for its growth. This bacterium can convert linoleic acid to CLA with the linoleate isomerase enzyme (5). Furthermore, it breaks down the proteins of the soybean meal, producing free amino acids and other compounds, with the substrate having more buffering capacity. On the other hand, fermentation of various carbohydrates in the substrate causes the production of lactic acid, reducing the pH value of the culture medium of *Bifidobacterium* (7). The 4% inoculation rate of *Bifidobacterium* in the SPC containing 70% humidity and incubated at 37 ° C showed the highest pH reduction, producing 9*cis* and 11*trans* CLA (c9-t11 CLA) and EPA at 0.18 and 0.39% of the total FA, respectively (Table 4). Meanwhile, the other isomers of CLA such as t10-c12 CLA were not detected (47). Moreover, *B. lactisis*, as other lactic acid bacteria such as *Lactobacillus plantarum* (42)), can grow on the SSF SPC. The soybean meal pressed cake has sufficient nutrient levels (13) to support the growth of these bacteria without the need to add carbohydrate and protein supplements (7, 9). Another researcher has also mentioned that the FAs proportion and their isomer types are dependent on pH, temperature, microbial inoculation level, substrate concentration and activation method used for bacterial strain (48).

**TABLE 4:** Fatty acids composition (% total fatty acids) of the SPC with (B) and without *Bifidobacter lactis* (A).

Fatty acids	A	B	Inoculation change (Δ*)
C12:0	1.55	1.27	−0.28
C14:0	1.26	1.73	+0.47
C14:1	0.41	0.3	−0.11
C15:0	0.26	1	+0.74
C15:1	0.29	0.18	−0.11
C16:0	18.57	29.3	+10.37
C16:1	0.21	0.23	+0.2
C17:0	0.22	0.3	+0.8
C17:1	ND	0.11	+0.11
C18:0	13.35	24.81	+11.35
C18:1t	0.34	0.22	−0.12
C18:1c	27.45	14.82	−12.63
C18:2t	0.29	0.31	+0.2
C18:2c	30.41	19.89	−10.52
C18:3t	ND	0.4	+0.4
C18:3c	1.92	1.86	−0.6
C20:0	0.68	0.56	−0.12
**CLA c9t11**	ND	0.18	+0.18
CLA t10c12	ND	ND	ND
C20:1	0.13	ND	−0.13
C20:2	ND	0.1	+0.1
C20:4n-6	ND	ND	ND
C22:0	0.51	0.44	−0.7
C22:1	ND	0.35	+0.35
**C20:5 n-3(EPA)**	ND	0.39	+0.39
C24:0	0.56	0.2	−0.54
C24:1	0.07	ND	−0.07
C22:6 n-3 (DHA)	ND	ND	ND

*Δ = B−A, ND: not detectable.

## Conclusion

Using microbial cultures in producing and increasing CLA and EPA concentrations in fermented foods is not an easy task and usually of meager yield. Despite the lower potency of *B. lactis* (BBo4, Persian Type, Culture Collection, Iran), as compared to other bifidobacteria strains, due to the value of pure isomer produced, this study was performed to evaluate the potency of the SPC as an almost oil-free substrate, considering SPC as one of the most source for bioactive compounds (26) extracted oil may contain these healthy materials although the huge waste of soybean oil production can supply the cheap and safe substrate for CLA and EPA production (37). The use of soybean meal increased lactic acid during solid-state fermentation. Due to the intrinsic properties of soybean meal, it increased the substrate fatty acids, thus producing conjugated linoleic and eicosapentaenoic acids. Given the experimental design conditions and methods adopted in the production of cheap isomers at a low cost, by considering the health features and probiotic aspects of the *B. lactis* strain, the soybean meal could be considered as a natural (plant) substrate available for producing this substance. While the findings here are valuable, more in-depth studies should be run in regard to the type of sub-bedding produced by the microbial strain through considering dietary supplements for the higher concentrations of CLA. It was revealed that the oil produced from plant waste could be adopted and the microbial isomerization method could be used to produce bioactive compounds and to make valuable substances; in addition to reducing the food production costs, it could decrease such outlets’ expenses. This, in turn, would improve the added value therein. At this age of recycling waste, in regard to healthy foods, resorting to natural waste products is of importance, in addition to protecting the environment.

In this context, the previous research has mostly focused on identifying CLA/CLNA-producing bacteria, evaluating production under general growth conditions, and using substrate concentration by chromatography/spectrophotometry through Bifidobacteria on soybean. The capability of some species of LAB including propionibacteria and bifidobacteria to in-vitro conjugate the LA or LNA has been shown over the years. Producing functional foods enriched in conjugated fatty acids by using it as a starter or adjunct culture can be considered a promising topic to for further development and study (38).

## Data Availability Statement

The original contributions presented in this study are included in the article/supplementary material, further inquiries can be directed to the corresponding author.

## Author Contributions

SA was mainly responsible for all phases of this research. MH developed the idea and guided the whole project and conducted analyses. ZM helped in the microbial analysis. HK and SJ also helped to improve the research and the manuscript. All authors contributed to the article and approved the submitted version.

## Conflict of Interest

The authors declare that the research was conducted in the absence of any commercial or financial relationships that could be construed as a potential conflict of interest.

## Publisher’s Note

All claims expressed in this article are solely those of the authors and do not necessarily represent those of their affiliated organizations, or those of the publisher, the editors and the reviewers. Any product that may be evaluated in this article, or claim that may be made by its manufacturer, is not guaranteed or endorsed by the publisher.
